# A cross-sectional study of student empathy across four medical schools in Denmark—associations between empathy level and age, sex, specialty preferences and motivation

**DOI:** 10.1186/s12909-022-03532-2

**Published:** 2022-06-23

**Authors:** Elisabeth Assing Hvidt, Jens Søndergaard, Sonja Wehberg, Niels Christian Hvidt, Christina Maar Andersen

**Affiliations:** 1 Research Unit for General Practice, Department of Public Health, University of Southern Denmark, J.B. Winsløwsvej 9 A, 5000 Odense, Denmark; 2 Department for Psychology, University of Southern Denmark, Odense, Denmark

**Keywords:** Empathy level, Cross-sectional study, Jefferson Empathy Scale (Student Version), Denmark, Medical education, Specialty preferences, Motivations

## Abstract

**Background:**

Professional empathy has been associated with a range of positive patient- and clinician outcomes and is therefore considered important to develop for future physicians. Measuring changes in empathy scores among medical students by using the Jefferson Scale of Empathy (Student version) (JSE-S) has led to mixed results. So far, no investigation of Danish medical students’ empathy development has been conducted. The aim of this study was therefore to examine the associations between empathy scores among Danish medical students and medical school, year of curriculum, age, sex, co-habitation, and parental status, specialty preferences and motivations for choosing medicine as a future profession.

**Methods:**

This was a cross-sectional questionnaire study. All medical students from four medical schools in Denmark in their first, third and sixth year (*N* = 4,178) were invited to participate in the study in October 2020. The associations between JSE-S sum score and the above explanatory factors were analysed by uni- and multivariable linear regression models.

**Results:**

The JSE-S was completed by 672 medical students. The overall mean score was 112.7. There were no statistically significant differences in empathy between medical schools, first, third- and sixth- year medical students, age groups or parental status. Female students and students living with a spouse or partner scored higher on JSE-S than male students or students living alone, and the sex difference remained statistically significant in the multivariable regression. In both the univariable and multivariable setting, preference for future medical specialty was statistically significant, with a decrease in scores for students choosing surgery-specialties. Motivational factors were not statistically significantly associated with empathy, although there was a slight upwards trend for one of the motivational categories, named “personal experiences”.

**Conclusions:**

Overall, our results showed neither decrease nor increase but instead rather stable empathy scores across years of curriculum of medical students in Denmark, adding to the mixed picture of empathy development among medical students. Our findings are consistent with positive associations found in international studies between empathy scores and higher age, female sex, specialty preferences for psychiatry and general practice and altruistic motivations for choosing to enroll. Although specialty preferences are changing during medical education, they may be used meaningfully as predictors of individual student empathy levels.

**Supplementary Information:**

The online version contains supplementary material available at 10.1186/s12909-022-03532-2.

## Background

Professional empathy is considered an important attribute and competence to have and further develop for students in the medical education [1]. Defined in the medical literature as the ability to understand a patient’s suffering and concerns combined with an ability to communicate this understanding and an intention to help [2–5], professional empathy has been associated with a number of beneficial patient and physician outcomes: more accurate diagnosis and treatment, increased patient satisfaction and compliance with treatment [3, 6–9], lower incidence of complaints and lawsuits, and lower levels of burnout and stress among physicians [2, 5, 10, 11]. Moreover, high scores on professional empathy among medical students are associated with increased satisfaction with their education, lower levels of stress and burnout, higher ratings of overall clinical competences given by medical school faculty, better interpersonal skills assessed by patients and greater teamwork skills [2, 5, 12–14].

Much attention has been dedicated across countries and cultures to investigate the development in empathy among medical students, relying largely upon the Jefferson Scale of Empathy (Student version) (JSE-S), a self-report scale developed specifically to measure professional empathy in respect to patient care, reflecting primarily the cognitive (as opposed to the emotional) dimension of empathy [1].

The vast body of literature on student empathy development from different parts of the world was recently reviewed by Andersen et al. [15], who found that results from most cross-sectional studies investigating empathy across year of medical curriculum show a decrease in empathy level, although stability and increase in empathy levels are also reported in some studies. These mixed results are possibly related to study limitations (differences between cohorts, lacking details of respondents) and differences in study designs (single-institutional, lack of control groups, etc.) [10]. In a more general sense, empathy varies in different cultures [16], and so the premise that empathy is comparable across medical educational cultures can be questioned. Taking these considerations to a side, results from studies in disparate cultural settings investigating associations between empathy scores and variables such as age, sex, and specialty preferences have largely found positive associations between empathy scores and higher age, female sex as well as student preferences for specialties that are more person and/or relationship-centred than technical/procedural [15, 17]. Lastly, studies have consistently found intrinsic motivational factors for studying medicine, e.g., a desire to care for patients, alleviate distress and save lives, to be positively associated with empathy in contrast to extrinsic motivational factors such as prestige, status, and future earning potential [18, 19]. Research investigating the influence of private life conditions—having children or not—on empathy is scarce. Since empathy constitutes an important psychosocial factor in parent–child-, and romantic relationships as well as human interaction in general [20], these social details of respondents are also important to take into consideration when investigating student empathy scores.

No investigation of Danish medical students’ empathy development and associations to the above-mentioned variables has yet been conducted. The aim of this study was to examine the associations between empathy scores as measured by JSE-S and students’ medical school, year of curriculum, age, sex, co-habitation, parental status, specialty preferences and motivations. In the light of the afore-mentioned studies, the following hypotheses are investigated:

1. There are no statistically significant differences in empathy scores across the four medical schools in Denmark since differences in culture and curriculum are expected to be small within the study sample.
2. Students in lower year of curriculum, i.e., 1^st^ year, will have a higher level of empathy than students of higher years of curriculum, i.e., 3^rd^ and 6^th^ year.
3. Older and female students score higher on empathy than their younger and male counterparts.
4. Students who have children and who live with others (i.e., with a partner and/or friends) score higher in empathy than those without children and/or living alone.
5. Students preferring specialties that are more person and/or relationship-centred than technical/procedural will score higher than those preferring technical/procedural specialties.
6. Students who choose to study medicine due to intrinsic, prosocial or altruistic motivations (helping other people or society) score higher on empathy compared to students motivated by prestige, status, or monetary gain.

## Methods/design

The study was designed as a national, cross-sectional questionnaire study targeting first, third- and sixth- year medical students in Denmark in October 2020.

### Study settings and samples

First, third- and sixth year medical students from all four medical schools in Denmark (University of Copenhagen (KU), Aarhus University (AU), University of Southern Denmark (SDU) and Aalborg University (AAU)) were included in the study. In terms of length and content of curriculum the four medical schools in Denmark are quite similar. Medical education in Denmark is standardized to last 6 years and divided into a three-year bachelor medical education and a three-year graduate medical education. Generally, the bachelor comprises basic biomedical science courses in the pre-clinical years (1^st^ and 2^nd^ year) and clinical clerkships towards the end of the bachelor (3^rd^ year), continuing in the graduate medical education with an increase in clinical student participation -and responsibility.

While the curriculum content in regard to medical humanities and empathy-enhancing subjects differ across universities (e.g., at some of the universities early contact with patients/citizens thought to cultivate empathy is included, at other universities literary courses like “narrative medicine” or medical ethics), teaching in communication (theory) and communication skills training (practice) through simulation (actors playing the role of patients) is included throughout the medical educations, covering approximately the same amount of European Credit Transfer System (ECTS) across medical schools.

In Denmark, the publicly funded universities offer tuition-free medical education thus reducing the influence of socioeconomic factors on medical students’ choice of education. In addition, the Danish State offer financial aid to every Dane over the age of 18 who choose to educate him-/herself further – regardless of socioeconomic standing. All study programs have a limited number of study places that are divided into two quotas, which have different admission procedures and requirements. In quota 1, the places are distributed based on grade point averages. The study places go to the applicants with the highest grades until there are no more quota 1 places, or until everyone is admitted. In quota 2, the places are distributed based on an admission test with one or more tests, including interviews. Here, the places go to the applicants who have passed the entrance exam best.

### Data collection

Data was collected from the 15^th^ of October to the 31^st^ of December 2020 through an online questionnaire, set up in the electronic survey system SurveyXact by Rambøll [21].

Students were informed about the study by receiving an information letter (the project was presented to students under the title: “Students’ relations, values and mental health – what are the associations?”. In the information letter it was clarified how the data of the student was used (in accordance with the General Data Protection Regulation (GDPR)) and the contact information on the principal investigators (EAH, CMA and JS) was provided together with the contact information of the data protection officers of the faculty.

To increase the response rate for this project, we had planned to visit students at the four universities during key lectures and request from teachers to use a couple of minutes to present the project. This was however made impossible by the Covid-19 pandemic and lockdown periods where all university teaching was diverted to online teaching. We did, however, advertise the project on university websites and in online student magazines. Access to communication channels for advertisement of the survey was easiest at the authors’ institution (the University of Southern Denmark (SDU)) which might have resulted in differences in the recruitment of respondents between universities.

### Ethics

Complying with European data protection rules, the University of Southern Denmark (SDU) approved the data processing activities regarding this project, including permission to extract the students’ civil registration number (CPR) and other relevant background information such as year, study start, grades, country origin, and registered the project under [Journal no. 10.181]. The study was furthermore approved by the Research Ethics Committee (REC) of University of Southern Denmark (SDU) [Journal no. 20/5351].

### The questionnaire

In the questionnaire, students filled out the JSE-S [2] which in this study was used as a measure of professional (cognitive) empathy. It is a 20-item scale developed to specifically measure medical students’ personal orientation toward empathy in respect to patient care. For each item, the students’ response is measured on a 7-point Likert scale (1-7). Reversely coded items were: 1, 3, 6, 7, 8, 11, 12, 14, 18, 19. A total sum score was calculated as the sum of scores of the directly worded items plus the reverse score of the negatively worded items. If one or two items had missing values, then these were replaced by the mean score of other items.

JSE-S has been extensively validated internationally. The JSE-S total score ranges from 20 to 140, with higher values indicating a higher degree of empathy. In past studies, total scores among medical students have ranged from 115 to 123.1 and standard deviations ranged from 9.9 to 14.1 [1]. Before including the JSE-S in the questionnaire, it was translated into Danish according to the WHO’s guidelines [22] and cognitive interviews were conducted by EAH and CMA with ten Danish medical students [23].

In the questionnaire students also specified university, age, sex (categorized as 18–24 and 25 +), co-habitation status (whether living alone, with spouse/partner or with others), and whether they have children or not, preferences for various specialities (see details below, Table 1), and motivations behind choosing medicine (open-ended question, see details below and Table 2). An overview of the other scales included in the questionnaire can be found in Assing Hvidt et al. [5].

**Table 1:** Baseline characteristics of participants (*N* = 672) and all medical students in the target population (*N* = 4178). The *p*-values refer to a Chi-squared test between respondents and non-respondents, excluding potential missing values

Variable	Participants	Target population	Response rates and *p*-values
	**N (%)**	**N (%)**	
**All**	672 (100.0)	4178 (100.0)	16.1%
**University**			*p* < 0.001
AAU	64 (9.5)	406 (9.7)	15.8%
AU	213 (31.7)	1306 (31.3)	16.3%
KU	164 (24.4)	1552 (37.1)	10.6%
SDU	231 (34.4)	914 (21.9)	25.3%
**Sex**			*p* < 0.001
Male	165 (24.6)	1407 (33.7)	11.7%
Female	507 (75.4)	2771 (66.3)	18.3%
A**ge categories**			*p* = 0.30
18–24 years old	434 (64.6)	2610 (62.5)	16.6%
25 +	238 (35.4)	1544 (37.0)	15.4%
**Educational year**			*p* = 0.18
1st	280 (41.7)	1624 (38.9)	17.2%
3rd	212 (31.5)	1326 (31.7)	16.0%
6th	180 (26.8)	1228 (29.4)	14.7%
**Living situation**			
Alone	177 (26.3)		
With spouse/partner	238 (35.4)		
With others	257 (38.2)		
**Parental status: children**			
Yes	32 (4.8)		
No	640 (95.2)		
**Specialty preference**			
General practice	111 (16.5)		
Anesthesiology	46 (6.8)		
Dermatology and venerology	8 (1.2)		
Endocrinology	13 (1.9)		
Gynecology/obstetrics	52 (7.7)		
Infection medicine	10 (1.5)		
Cardiology	14 (2.1)		
Surgery	46 (6.8)		
Neurosurgery	14 (2.1)		
Neurology	19 (2.8)		
Oncology	8 (1.2)		
Ortopedic surgery	23 (3.4)		
Psychiatry	23 (3.4)		
Pediatrics	64 (9.5)		
Thoracic surgery, cardiac surgery	9 (1.3)		
Less frequent category, *N* < 10	67 (10.0)		
Missing	145 (21.6)

**Table 2:** Motivations for studying medicine (categorized) by explanatory factors. Percentages are row-wise. It was possible to state more than one motivation: *N* = 253 participants stated one motivational factor, *N* = 301 two, *N* = 50 three, *N* = 2 four and *N* = 66 none

		**Motivations for studying medicine**				
**Variable**	**N (100%)**	**Biology**	**Prestige**	**Helping others**	**Personal experiences**	**None**
**Overall**	672	412 (61.3)	90 (13.4)	411 (61.2)	100 (14.9)	66 (9.8)
**University**						
AAU	64	37 (57.8)	5 (7.8)	48 (75.0)	11 (17.2)	2 (3.1)
AU	213	144 (67.6)	29 (13.6)	132 (62.0)	31 (14.6)	21 (9.9)
KU	164	98 (59.8)	27 (16.5)	97 (59.1)	16 (9.8)	15 (9.1)
SDU	231	133 (57.6)	29 (12.6)	134 (58.0)	42 (18.2)	28 (12.1)
**Sex**						
Male	165	93 (56.4)	29 (17.6)	99 (60.0)	15 (9.1)	18 (10.9)
Female	507	319 (62.9)	61 (12.0)	312 (61.5)	85 (16.8)	48 (9.5)
**Age category**						
18–24 years old	434	273 (62.9)	54 (12.4)	264 (60.8)	63 (14.5)	44 (10.1)
25 +	238	139 (58.4)	36 (15.1)	147 (61.8)	37 (15.5)	22 (9.2)
**Educational year**						
1st	280	162 (57.9)	38 (13.6)	158 (56.4)	54 (19.3)	34 (12.1)
3rd	212	141 (66.5)	23 (10.8)	135 (63.7)	32 (15.1)	15 (7.1)
6th	180	109 (60.6)	29 (16.1)	118 (65.6)	14 (7.8)	17 (9.4)
**Living situation**						
Alone	177	94 (53.1)	18 (10.2)	96 (54.2)	32 (18.1)	21 (11.9)
With spouse/partner	238	149 (62.6)	28 (11.8)	151 (63.4)	35 (14.7)	23 (9.7)
With others	257	169 (65.8)	44 (17.1)	164 (63.8)	33 (12.8)	22 (8.6)
**Parental status: children**						
Yes	32	18 (56.3)	4 (12.5)	16 (50.0)	8 (25.0)	3 (9.4)
No	640	394 (61.6)	86 (13.4)	395 (61.7)	92 (14.4)	63 (9.8)

To gather information regarding specialty preferences, students were asked to choose one specialty from a drop-down menu of all available medical specialties in Denmark (see list of medical specialities, appendix Table S1). To gather information regarding student motivation for choosing to enrol, students were asked the following open question in the questionnaire: “What is your motivation for choosing the medical education?”, stimulating reflections rather than predefined answer categories.

The data on subjective motivations were imported into Excel, reviewed independently by EAH and CMA who made an inductive coding of each statement (e.g., sentences indicating that motivating the choice to enrol was obtaining monetary reward, people’s respect or a desire to care for patients were given the codes “money”, “status”, “helping others” respectively). Following this, EAH and CMA discussed the preliminary codes and how to cluster them into ten codes, e.g., by collapsing “money” and “prestige” and “status” into one code (see Table 2). Disagreements were resolved by discussions or by consulting another author (SW). The following four overall categorizations were subsequently developed in order to do statistical comparisons: 1) Biology, indicating that the biological and physiological aspects of medicine motivate the choice to enrol 2) Prestige, indicating that the prospect of future monetary gains, job security and societal status connected to the job motivate the choice to enrol 3) Helping others, indicating that altruistic and communicative interests motivate the choice to enrol and lastly 4) Personal experiences, indicating that prior good or bad experiences with illness (either through self or others), and a desire to optimize care, motivate the educational choice.

### Outcomes, explanatory factors and statistical analysis

The outcome was self-reported empathy sum scores as measured with JSE-S within the cross-sectional sample of medical students. Explanatory factors were: university, age, sex, co-habitation, parental status, and specialty preferences and motivations behind choice of study.

Data analysis were both uni- and multivariable linear regression models for the outcome measure JSE-S. Throughout the analyses, a *p*-value below 0.05 was considered statistically significant. In addition, we compared responders to target group with respect to university, educational year, age and sex by a Chi-squared test, excluding potential missing observations.

## Results

### Participant characteristics

Table 1 includes both an analysis of response rates and presents baseline characteristics of completers. A total of 4,178 medical students received an invitation to participate in the study of which 672 completed the questionnaire completely (16.1%), 215 only partially and 3,291 did not respond.

Response rates differed across universities and between sexes (*p*-values < 0.001) (Table 1), while there were no statistically significant differences in response rates according to age or educational years. Across universities, response rates ranged from 10.6% in Copenhagen to 25.3% at the University of Southern Denmark (SDU). Female students were more inclined to respond than male students (18.3 vs. 11.7%).

Looking at the distribution of characteristics in our analysis sample, participants were most often from the University of Southern Denmark (SDU) (34.4%) followed by Aarhus University (AU) (31.7%), University of Copenhagen (KU) (24.4%) and Aalborg University (AAU) (9.5%). Furthermore, participants were mostly female (75.4%), under the age of 25 years (64.6%) and first year students (41.7%) (Table 1). The participants were mostly co-habitants (73.6%) (living with others or spouse/partner versus living alone) and had no children (95.2%). The most frequently stated specialty preferences were family medicine/general practice (16.5%), pediatrics (9.5%) and gynaecology and obstetrics (7.7%).

### Associations between empathy and explanatory factors

Table 3 depicts the students’ score on JSE-S, showing the range of scores = 51–139, mean (SD) = 112.7 (10.8), interquartile range = 107–120, median = 113. The corresponding univariable regression results (Table 4) showed no statistically significant differences in empathy between universities, first, third- and sixth- year medical students, age groups or parental status. Female students scored 3.7 (95% confidence interval (CI): 1.8;5.5), *p*-value < 0.001 points higher on JSE-S than male students, and this difference remained statistically significant in the multivariable regression (2.5 (CI: 0.6;4.5), *p*-value < 0.012. Further, compared to students that live alone, those who live with a spouse or partner scored 2.8 (CI: 0.7;4.9), *p*-value = 0.0081 points higher on empathy. The same trend was observed in the multivariable model, though the difference was no longer statistically significant.

**Table 3:** Distribution of JSE-S empathy scores: mean and standard deviation (SD), median and interquartile range (IQR), and range (minimum–maximum)

Variable	N	Mean (SD)	Median (IQR)	Range
**All**	672	112.7 (10.8)	113.0 (106.7–120.0)	51.0 to 139.0
**University**				
AAU	64	112.8 (10.0)	113.0 (106.5–119.0)	91.0 to 134.0
AU	213	113.2 (10.0)	114.0 (107.0–120.0)	82.0 to 136.0
KU	164	112.6 (11.8)	113.0 (106.2–121.0)	51.0 to 139.0
SDU	231	112.3 (10.9)	112.0 (106.0–120.0)	66.0 to 134.0
**Sex**				
Male	165	110.0 (10.7)	111.0 (104.0–117.0)	66.0 to 131.0
Female	507	113.6 (10.6)	114.0 (107.0–121.0)	51.0 to 139.0
**Age categories**				
18–24 years old	434	112.5 (10.4)	113.0 (107.0–120.0)	66.0 to 136.0
25 +	238	113.1 (11.4)	114.0 (106.0–121.0)	51.0 to 139.0
**Educational year**				
1st	280	112.3 (11.6)	113.0 (106.0–120.0)	51.0 to 136.0
3rd	212	113.0 (9.5)	113.0 (108.0–119.0)	79.0 to 134.0
6th	180	113.1 (10.8)	113.3 (106.0–121.0)	82.0 to 139.0
**Living situation**				
Alone	177	111.4 (11.7)	112.0 (106.0–118.0)	51.0 to 134.0
With spouse/partner	238	114.2 (10.2)	114.0 (107.0–122.0)	82.0 to 139.0
With others	257	112.2 (10.5)	112.0 (107.0–119.0)	66.0 to 136.0
**Parental status: children**				
Yes	32	113.7 (11.2)	112.0 (108.0–121.5)	90.0 to 134.0
No	640	112.7 (10.8)	113.0 (106.2–120.0)	51.0 to 139.0
**Specialty preference**				
General practice	111	115.5 (8.8)	116.0 (110.0–121.0)	91.0 to 134.0
Anesthesiology	46	110.6 (10.0)	110.5 (105.0–117.0)	82.0 to 134.0
Dermatology and venerology	8	112.1 (7.7)	110.5 (108.0–118.5)	100.0 to 123.0
Endocrinology	13	113.3 (13.2)	112.0 (106.0–125.0)	84.0 to 130.0
Gynecology/obstetrics	52	114.6 (11.5)	115.5 (106.0–124.0)	93.0 to 136.0
Infection medicine	10	112.9 (8.8)	112.5 (110.0–121.0)	96.0 to 127.0
Cardiology	14	109.4 (14.5)	111.5 (98.0–120.0)	82.0 to 132.0
Surgery	46	108.7 (10.1)	111.0 (102.0–116.0)	83.0 to 126.0
Neurosurgery	14	108.3 (17.7)	115.0 (105.0–118.0)	51.0 to 120.0
Neurology	19	112.3 (9.5)	113.0 (104.0–117.0)	95.0 to 128.0
Oncology	8	112.6 (10.9)	113.0 (108.5–116.5)	93.0 to 132.0
Ortopedic surgery	23	109.9 (13.5)	113.0 (105.0–118.0)	66.0 to 128.0
Psychiatry	23	119.7 (9.5)	122.0 (114.0–127.0)	97.0 to 131.0
Pediatrics	64	116.6 (8.8)	118.0 (109.5–123.0)	98.0 to 139.0
Thoracic surgery, cardiac surgery	9	103.7 (11.7)	109.0 (93.0–114.0)	88.0 to 117.0
Less frequent category, *N* < 10	67	111.0 (11.5)	109.0 (104.0–119.0)	66.0 to 133.0
Missing	145	111.6 (10.1)	112.0 (105.3–118.0)	79.0 to 133.0
**Motivations**				
Biology (yes)	412	112.9 (10.0)	113.0 (107.0–120.0)	66.0 to 136.0
Prestige (yes)	90	112.0 (10.2)	112.0 (106.0–119.0)	88.0 to 134.0
Helping others (yes)	411	113.0 (10.4)	113.0 (107.0–120.0)	51.0 to 139.0
Personal experiences (yes)	100	114.7 (10.6)	116.0 (110.0–122.0)	66.0 to 132.0
At least one motivation stated	606	113.1 (10.7)	113.3 (107.0–120.0)	51.0 to 139.0
None stated	66	109.4 (11.3)	110.0 (103.0–118.0)	79.0 to 134.0

**Table 4:** Estimated regression coefficients and corresponding 95% confidence intervals in parentheses from both univariable and multivariable linear regression models (*N* = 672). Overall Wald-test *p*-values for categories with more than two levels are in italics

	**Univariable models**		**Multivariable model**	
**Variable**	**Estimate (95% CI)**	***p*-value**	**Estimate (95% CI)**	***p*-value**
**Motivations**				
Biology (yes)	0.4 (-1.3;2.1)	0.6348	0.0 (-1.9;2.0)	0.98
Prestige (yes)	-0.8 (-3.2;1.6)	0.5027	-0.6 (-3.0;1.9)	0.64
Helping others (yes)	0.8 (-0.9;2.4)	0.3731	-0.5 (-2.5;1.5)	0.62
Personal experiences (yes)	2.3 (0.0;4.6)	0.0459	2.1 (-0.5;4.8)	0.11
None stated	-3.6 (-6.4;-0.9)	0.0092	-4.1 (-7.6;-0.5)	0.027
**University**		*0.8444*		*0.96*
AAU	Ref		Ref	
AU	0.4 (-2.6;3.4)	0.8079	0.6 (-2.4;3.7)	0.69
KU	-0.2 (-3.4;2.9)	0.8776	0.5 (-2.6;3.6)	0.76
SDU	-0.5 (-3.5;2.5)	0.7249	0.1 (-2.9;3.1)	0.94
**Female Sex**	3.7 (1.8;5.5)	< 0.001	2.5 (0.6;4.5)	0.012
**Age categories**				
18–24 years old	Ref		Ref	
25 +	0.6 (-1.1;2.3)	0.4809	0.2 (-2.4;2.9)	0.86
**Educational year**		*0.6359*		*0.77*
1st	Ref		Ref	
3rd	0.8 (-1.2;2.7)	0.4325	0.7 (-1.3;2.6)	0.49
6th	0.8 (-1.2;2.9)	0.4167	0.1 (-2.9;3.2)	0.93
**Living situation**		*0.0195*		*0.26*
Alone	Ref		Ref	
With spouse/partner	2.8 (0.7;4.9)	0.0081	1.8 (-0.4;4.0)	0.11
With others	0.8 (-1.2;2.9)	0.4287	0.6 (-1.5;2.8)	0.57
**Parental status: children**				
Yes	Ref		Ref	
No	-1.0 (-4.8;2.8)	0.6039	0.8 (-3.1;4.8)	0.68
**Specialty preference**		*< 0.001*		*< 0.001*
General practice	Ref		Ref	
Anesthesiology	-4.9 (-8.5;-1.3)	0.0083	-3.4 (-7.1;0.3)	0.074
Dermatology and venerology	-3.4 (-10.9;4.2)	0.3800	-4.1 (-11.6;3.4)	0.28
Endocrinology	-2.2 (-8.3;3.8)	0.4651	-1.3 (-7.4;4.8)	0.67
Gynecology/obstetrics	-0.9 (-4.3;2.6)	0.6185	-1.5 (-5.0;2.0)	0.40
Infection medicine	-2.6 (-9.4;4.2)	0.4533	-2.3 (-9.1;4.6)	0.52
Cardiology	-6.1 (-11.9;-0.2)	0.0415	-4.7 (-10.6;1.2)	0.12
Surgery	-6.8 (-10.4;-3.1)	< 0.001	-6.3 (-10.0;-2.7)	< 0.001
Neurosurgery	-7.2 (-13.0;-1.3)	0.0162	-7.4 (-13.3;-1.5)	0.014
Neurology	-3.2 (-8.3;1.9)	0.2221	-2.7 (-7.8;2.5)	0.31
Oncology	-2.9 (-10.4;4.7)	0.4545	-3.0 (-10.6;4.6)	0.44
Ortopedic surgery	-5.6 (-10.3;-0.9)	0.0204	-4.4 (-9.2;0.3)	0.068
Psychiatry	4.2 (-0.5;8.9)	0.0791	5.4 (0.6;10.2)	0.027
Pediatrics	1.1 (-2.1;4.4)	0.4875	1.6 (-1.7;4.9)	0.35
Thoracic surgery, cardiac surgery	-11.8 (-19.0;-4.7)	0.0012	-11.3 (-18.5;-4.1)	0.002
Less frequent category, *N* < 10	-4.5 (-7.7;-1.3)	0.0056	-3.7 (-6.9;-0.4)	0.026
Missing	-3.9 (-6.5;-1.3)	0.0032	-3.3 (-6.0;-0.6)	0.016
**Constant**			111.2 (105.4;117.1)	< 0.001

In both the univariable and multivariable setting, preference for future medical specialty was statistically significant with an overall *p*-value < 0.001. In the multivariable model, with the specialty general practice as reference, students choosing psychiatry as specialty preference scored 5.4 (CI: 0,6;10.2), *p*-value = 0.027 higher, whereas a decrease in scores was found for those choosing surgery -6.3 (CI: -10.0;-2.7), *p*-value < 0.001, neurosurgery -7.4 (CI: -13.3;-1.5), *p*-value = 0.014 and cardiothoracic surgery -11.3 (CI: -18.5;-4.1), *p*-value = 0.002, see Table 4.

Concerning the different motivational aspects, there were no statistically significant differences in the multivariable model except for “no motivation stated”, which was associated with a decrease in empathy of -4.1 (CI: -7.6;-0.5), *p*-value = 0.027. The estimated coefficients were around zero for “interest in human body” (biology), “prestige and helping others/society”. The motivation of “personal experiences” was associated with a slightly larger empathy score of 2.1 (CI: -0.5;4.8), which was comparable to the effect of living status, though both were not statistically significantly different from zero.

### Participants’ motivation

**Fig. 1:**
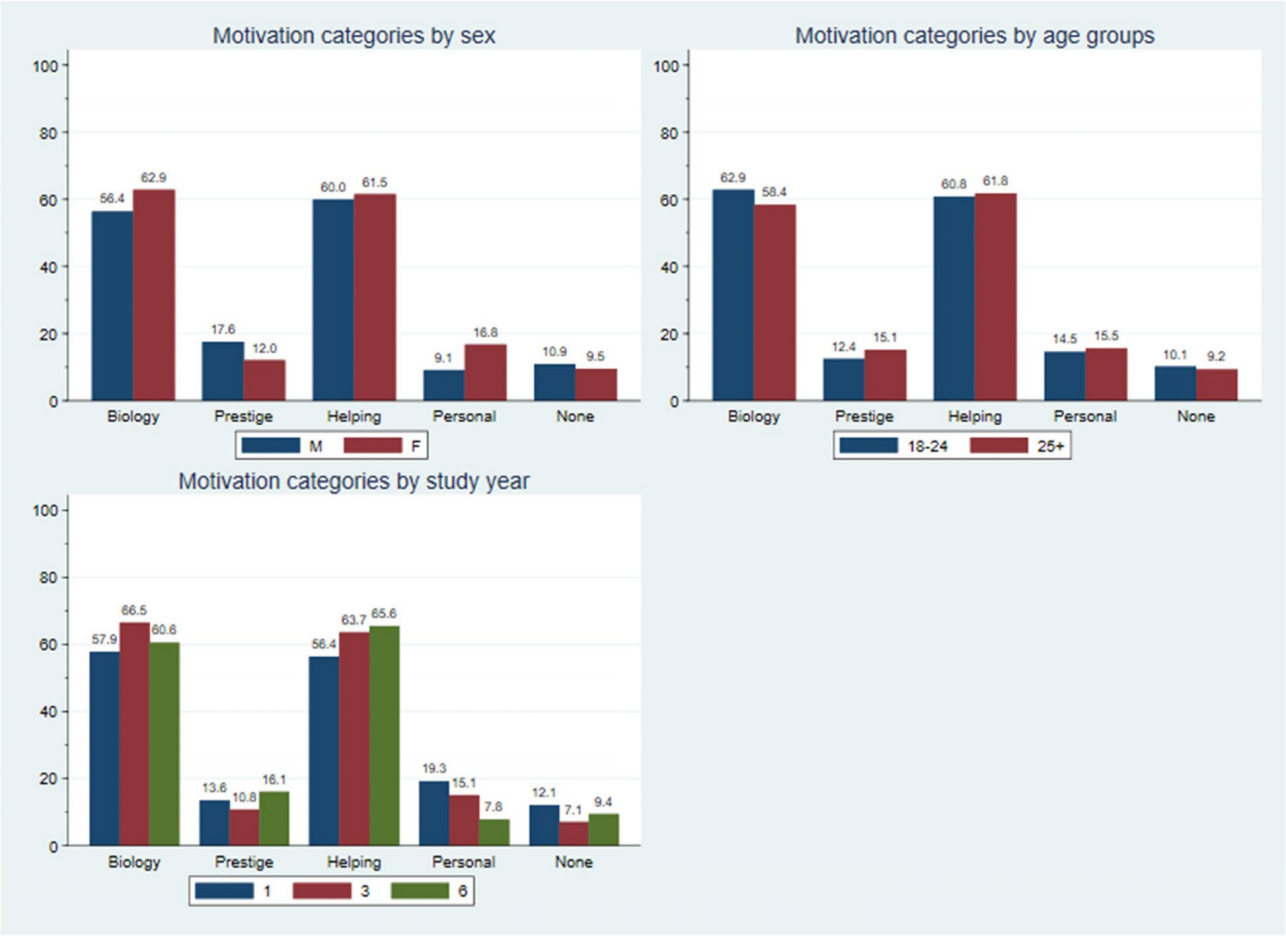
Motivations for studying medicine (categorized) by sex, age groups and educational (study) year

Table 2 and Fig. 1 depict student motivations. Among all the students the motivations most often given (students could indicate as many motivations as they would like) were interest in the human body/biology (61.3%) and interest in helping others/society (61.2%). When comparing motivational categories among students at different years of curriculum, and among those still enrolled, the category to help others/society increased with higher educational year ((1^st^ (56.4%), 3^rd^ (63.7%) and 6^th^ 65.6%)) whereas the category personal experience decreased with higher year of curriculum (1^st^ (19.3%), 3^rd^ (15.1%) and 6^th^ (7.8%)). More male students were included in the category prestige than were female students (17.6% vs 12.0%) whereas more female students were included in the category personal experiences compared to male students (16.8% vs 9.1%).

## Discussion

### Universities, age, sex, living status

One of our hypotheses: that we would find no statistically significant differences in empathy across medical schools in Denmark has been confirmed by our study results showing relatively high and stable empathy scores across all four medical schools. The results showing empathy scores to be stable across educational years (and age) are consistent with results found in other studies [24–27]. Explanations for why empathy scores surprisingly did not decrease from first to sixth-year medical students (as hypothesized and found in prior evidence, see [15]) might be that clinically-situated empathy has been highlighted in recent years as an integral part of medical professionalism [1, 28, 29], and thus formally as a “need to have” among medical students. This has led universities to include various programmes in the medical curriculum intended to foster empathy, such as reflective writing, literature courses, theatre workshops, communication skills training, etc. [30, 31]. Although several methodological limitations exist in studies evaluating the impact of these interventions on students’ empathy, research suggests that focused educational interventions may be successful at fostering and maintaining empathy in medical students [32]. Having said this, it must be noted that with the present study’s cross-sectional design, one cannot know whether decreasing and/or increasing empathy scores might in fact have led to dropouts, meaning that the results of this study only reflect the development in empathy among those students who have chosen to stay enrolled up until the last year of curriculum.

In concert with many other studies recently reviewed in Andersen et al. [15], and supporting our hypothesis relating to associations between empathy and sex, we found that female medical students scored higher on empathy compared to male medical students. Explanations for this dominating trend can be found within evolutionary psychology where empathy is viewed as a skill that women have developed due to their evolutionary primary role as caretakers of children, the elderly and sick people whereas men have developed skills related to hunting and defending the tribe [33]. This view is also supported by the fact that most professional caretaker positions are occupied by women [34, 35]. Also, as pointed out by some authors who raise attention to the influence of socio-cultural norms and traditions in respect to what can appear as a constraining collective and societal shaping of women’s identity, empathic behavior is expected of women by society—a social and cultural construction of female personality traits, including conscientiousness, openness and agreeableness – a social desirability that we might see reflected in female respondents’ self-reporting empathy scores [36].

### Specialties and motivations

The findings did support the hypothesis that students preferring specialties that are more person and/or relationship-centred than technical/procedural specialties would score higher on the empathy scale than those preferring technical/procedural specialties. This hypothesis seemed logical since technical/procedural specialties have been characterized as involving a relatively low degree of relational contact and a low level of interpersonal continuity [37]. This suggests that students answer consistently between specialty preferences and empathy orientations (presented in the JSE-S) which might mean that asking students about their specialty preferences at different time points (to capture possible changes of preferences) has a predictive validity, possibly higher than the one of a self-report empathy questionnaire such as JSE-S.

In relation to the specialties psychiatry and general practice, we also found that students indicating psychiatry as a specialty preference scored higher on empathy than those students choosing general practice, although scores in both groups were high. The difference in empathy scores between the two groups of students can only be speculated upon. Although some psychiatrists go into the profession because of their interest in mental diseases, others might be particularly driven by moral and social indignation and compassion towards those who are stigmatized and live at the margins of society. General practice as specialty involves contact with a large proportion of healthy people coming to see the doctors about common, minor and/or self-limiting, medical conditions. Treating a relatively high number of people with chronic conditions, general practitioners often develop an ongoing relationship with their patients, providing continuity of care. Finally, the findings did support the hypothesis that students who choose to study medicine due to prosocial or altruistic motivations (helping other people or society) score higher on empathy compared to students motivated by prestige, status, or monetary gain. Educators can foster altruistic motivations among students by addressing altruism-enhancing patient-centred illness aspects as strived for in the humanities subjects, e.g., health psychology and narrative medicine. Supporting this view, neurobiological studies show that motivations modulate empathy at the neural level [38]. For example, having a desire to help other people who are in need and alleviate their suffering makes people especially motivated to empathize, which increases empathy-related brain activity. Conversely, the desire for prestige, status and monetary gain makes people empathy avoidant, since showing empathy might be considered time consuming and non-rewarding, and hence decrease empathy-related brain activity [38, 39].

Moreover, we found that among those students still enrolled motivations among the students who were motivated by a wish to help others seemed to increase with year of curriculum. Although this observed tendency needs to be confirmed through longitudinal data, it can be explained by an increase in clinical experiences and patient contact over the years of curriculum that might lead to experiences of making a difference and causing positive changes via one’s work competences. Observing the effect of one’s work, whether on a biomedical or psycho-social level, might increase one’s altruistic motivation.

The percentage of students (based on those remaining) expressing the motivations grouped under the category “personal experience” was also found to decrease. This category, indicating a desire to optimize care, might have been dominant behind the choice to enroll but might have diminished as other educational input and experiences are received. The decrease in motivations grouped under the category “personal experience” might also be caused by feelings of disillusion and/or realizing that the system is too powerful to be changed.

Interestingly, in relation to both specialty preferences and motivations, we found that there were no statistically significant differences with respect to motivation in the multivariable regression model but considerable effects with respect to specialty preferences. This might suggest that specialty preferences (which are purely a wish at a certain time point) has a stronger link to (latent) empathy level than their current (retrospective) motivation. It could be interesting to investigate the hypothesis that degree of technical/procedural elements in a specialty is associated with empathy score.

### Strengths and limitations

In our survey study, we invited the total population of all first, third- and sixth- year medical students in Denmark instead of a sample. Being a multi-institutional study, the findings are more transferrable to similar educational contexts than studies made in a single institution. As is always the case with surveys, the self-selected participants might have been particularly interested in the subject at hand, possibly also displaying a relatively higher level of empathy than those who did not contribute. A further limitation of this study is the relatively low response rate of 16%. Low response rates in student surveys in higher education, in particular in connection with student evaluations of teaching, are however a well-known issue [40]. Added to this, students might have felt a digital survey fatigue since a general trend during the Covid-19 pandemic was that students felt over-surveyed [41]. A further limitation of this study is the self-report measures employed to measure empathy. Research has however shown that statistically significant associations exist between students’ self-reported empathy (JSE-S) and simulated patients’ evaluations of students’ empathy [14]. Furthermore, there might have been a recollection bias in play in relation to students’ motivations for enrolling in medical education, in particular for third and sixth- year students.

## Conclusion

Our results show that empathy scores did not decrease or increase from first to sixth-year Danish medical students. Overall, our study results, showing high and stable empathy scores across years of curriculum of medical students in Denmark, add to the mixed picture that a large body of cross-sectional studies have conveyed. Longitudinal designs, albeit proven difficult to establish worldwide, are therefore still needed to gain a deeper insight in how empathy levels develop over time in the medical education in Denmark and elsewhere. There were no statistically significant differences in empathy between universities, first, third- and sixth- year medical students, age groups, parental status or motivations. Our findings are consistent with statistically significant associations found in international studies between empathy scores (measured by JSE-S), female sex and person and/or relation-centred specialty preferences. Although specialty preferences are likely to change during a medical education, and all medical specialities ideally involve engaging in empathic understanding of the patient’s life circumstances, they may be used meaningfully as predictors of individual student empathy levels.

## Supplementary Information

**Additional file 1: Table S1. **Students’ medical specialties preferences based on all available questionnaire answers (*N*=844, partially completed questionnaire data included for *N*=174). From the *N*=672 participants, *N*=2 did not answer the question on specialty preference. For analysis, categories which included less than 10 students were gathered into a common category “Less frequent category, *N*<10”. The following specialties were not chosen once: Occupational medicine, Clinical pharmacology, Clinical physiology and nuclear medicine, Classical thoracic surgery.

## Data Availability

The datasets generated and/or analyzed during the current study are available from the corresponding author on reasonable request.

## Abbreviation

JSE-S: The Jefferson Scale of Empathy – Student Version
