# Supplementary material for: A cross-sectional study of student empathy across four medical schools in Denmark—associations between empathy level and age, sex, specialty preferences and motivation
Source: BMC Med Educ. 2022 Jun 23;22:489. doi: 10.1186/s12909-022-03532-2 (PMC9223265; doi:10.1186/s12909-022-03532-2)
Supplement: Supplementary file 1 — Additional file 1: Table S1. Students’ medical specialties preferences based on all available questionnaire answers (N=844, partially completed questionnaire data included for N=174). From the N=672 participants, N=2 did not answer the question on specialty preference. For analysis, categories which included less than 10 students were gathered into a common category “Less frequent category, N<10”. The following specialties were not chosen once: Occupational medicine, Clinical pharmacology, Clinical physiology and nuclear medicine, Classical thoracic surgery. [file 12909_2022_3532_MOESM1_ESM.docx]

Supplementary Table S1: Students’ medical specialties preferences based on all available questionnaire answers (N=844, partially completed questionnaire data included for N=174). From the N=672 participants, N=2 did not answer the question on specialty preference. For analysis, categories which included less than 10 students were gathered into a common category “Less frequent category, N<10”. The following specialties were not chosen once: Occupational medicine, Clinical pharmacology, Clinical physiology and nuclear medicine, Classical thoracic surgery.

| **Specialty (answer category)** | **Comment** | **N of 844** |
| --- | --- | --- |
| Do not know | Combined with missing category | 171 |
| General practice |  | 141 |
| Pediatrics |  | 82 |
| Gynecology/obstetrics |  | 62 |
| Surgery |  | 61 |
| Anesthesiology |  | 61 |
| Psychiatry |  | 34 |
| Orthopedic surgery |  | 29 |
| Neurology |  | 27 |
| Neurosurgery |  | 17 |
| Endocrinology |  | 16 |
| Infection medicine |  | 15 |
| Cardiology |  | 15 |
| Dermatology and venereology |  | 12 |
| Thoracic surgery/cardiac surgery |  | 11 |
| Oncology |  | 10 |
| Forensic medicine | Less frequent category, N<10 | 9 |
| Clinical genetics | Less frequent category, N<10 | 8 |
| Ear-nose-throat surgery | Less frequent category, N<10 | 7 |
| Ophthalmology | Less frequent category, N<10 | 6 |
| Child and adolescent psychiatry | Less frequent category, N<10 | 6 |
| Plastic surgery | Less frequent category, N<10 | 6 |
| Gastro-enterology | Less frequent category, N<10 | 5 |
| Diagnostic radiology | Less frequent category, N<10 | 4 |
| Hematology | Less frequent category, N<10 | 4 |
| Nephrology | Less frequent category, N<10 | 4 |
| Vascular surgery | Less frequent category, N<10 | 4 |
| Pulmonary medicine | Less frequent category, N<10 | 3 |
| Rheumatology | Less frequent category, N<10 | 3 |
| Urology | Less frequent category, N<10 | 3 |
| Pathological anatomy - pathology | Less frequent category, N<10 | 2 |
| Clinical biochemistry | Less frequent category, N<10 | 2 |
| Clinical immunology | Less frequent category, N<10 | 1 |
| Geriatrics | Less frequent category, N<10 | 1 |
| Social medicine/community medicine | Less frequent category, N<10 | 1 |
| Clinical microbiology | Less frequent category, N<10 | 1 |
